# Supplementary material for: Are carbon emissions trading and green financial instruments synergistic? -Comprehensive quantitative research based on content analysis
Source: PLoS One. 2024 Mar 7;19(3):e0298601. doi: 10.1371/journal.pone.0298601 (PMC10919601; doi:10.1371/journal.pone.0298601)
Supplement: S1 Data — (ZIP) [file pone.0298601.s001.zip › Data/地级市Z_F工作报告.zip!/╣π╬≈/╖└│╟╕█╩╨/└┤╫╘╣1⁄2╓┌║┼í╛┬φ┐╦╩2╛▌═°í┐_2017.docx]

各位代表：

现在，我代表市人民政府向大会作政府工作报告，请予审议，并请市政协各位委员提出意见。

一、2016年工作回顾

过去一年，面对复杂的发展环境，在自治区党委、政府和市委的坚强领导下，在市人大、市政协的监督支持下，我们紧紧依靠全市各族人民，牢固树立新发展理念，以边海经济带建设为主线，发挥东兴试验区等国家级开放平台作用，主动作为，克难攻坚，统筹稳增长和推进供给侧结构性改革，扎实推进产业转型升级、基础设施建设、农村全面脱贫、教育卫生四大攻坚战，经济呈现稳中向好的发展态势，社会保持和谐稳定，实现了“十三五”良好开局。

——主要经济指标均衡高速增长。预计全市生产总值676.1亿元，增长9.1%，增速全区第二；固定资产投资600.1亿元，增长14.1%；规上工业总产值1501.2亿元，增长14.3%，增速全区第一；财政收入75.6亿元，增长7%；招商引资内资项目到位资金483.2亿元，增长10.4%，实际利用外资5.1亿美元，增长11%；社会消费品零售总额111.9亿元，增长10.8%；外贸进出口总额587.8亿元，增长10%，总额和增速均排全区第二。

——一批重大项目取得新进展。十大龙头产业项目带动作用日益凸显，红沙核电二期工程4号机组开工，钢铁基地高端冷轧钢板顺利下线，中电二期全面建成，枫叶粮油、盛隆焦化二期投产，中海油LNG储运、科元新材料等重大项目加快推进。

——先行先试效应持续扩大。国家开放型经济新体制试点试验四大改革和六大探索取得实质性成效。东兴试验区跨境劳务合作试点扎实推进，东盟货币服务平台、跨境保险服务中心、跨境人民币贷款和个人本外币兑换特许业务等多项先行先试成果不断巩固和扩大。东兴-芒街跨境自驾游正式开通，东兴陆路进境水果口岸获批。上线运行西部首个沿海口岸国际贸易“单一窗口”。

——工业质量不断增强。全市规上工业增加值完成332.8亿元，增长12.4%，增速全区第一。企业技术改造投资225亿元，增长96.2%，增速全区第一。产值超亿元的规上工业企业124家，其中百亿元企业3家，十亿元企业26家。新增广西工业质量标杆企业3家。规上万元工业增加值能耗下降4.4%。

——民生福祉稳步提升。全市民生支出99.2亿元，占财政支出的78.1%，为民办实事十大工程项目基本完成。城市、农村低保月人均补助分别上升到306元、140元。城镇居民人均可支配收入29758元，增长7.9%，农村居民人均可支配收入12113元，增长10.2%。城镇登记失业率1.7%。居民生活用气每立方售价由4.8元下调至3.21元，为全区最低。注重环境生态保护，主要水源水质优良率100%，城市空气质量优良率99.5%，稳居全区第一。

过去一年，我们主要抓了十个方面工作：

（一）全力以赴稳增长。全面落实国务院和自治区一系列政策措施，确保经济稳定运行。坚持抓早抓好抓实，年初就将年度各项目标任务分解落实到各责任单位，细化分解任务，抓好工作落实。围绕自治区提出“六稳”要求，通过降低工业企业成本，增强企业盈利能力和发展活力；实施“边海经济带工程包”，启动海湾新区建设，稳定投资增速；抓好商贸物流、特色旅游、电商等服务业发展，提高消费档次和消费量；发展“特色、精致、高效、品牌”农业，提升农业效益；提高通关便利化水平，推动进出口贸易逆势上扬；落实营改增等减税政策，盘活存量资金，实现财政平稳运行。

（二）克难攻坚推项目。实施“边海经济带工程包”项目709项，其中全年新开工249项。强化项目保障，完成征地面积1.65万亩，新增建设用地、用海指标1.78万亩。筹措项目建设资金，成功申报中央预算内投资项目108个，策划PPP项目一批，发展后劲和动力不断增强。

（三）多措并举优供给。积极推动供给侧结构性改革，“三去一降一补”工作取得明显成效。落实自治区降成本41条措施，出台46条配套细则，清理涉企收费，为企业减负5亿多元。优化传统产业供给，实施产业转型升级，推进有色金属、磷化、制糖等产业“二次创业”。推动房地产去库存，出台《防城港市农民工等新市民购房补助细则》、棚户区改造货币化安置等措施，开展口岸联检大楼“房地产+港航服务”试点，全年销售商品房面积200多万平方米。稳妥去杠杆防风险，实施政府债务限额管理，申报置换债券23.97亿元，提前偿还高利率债务11.16亿元。加强产业投资基金运营管理，设立产业基础设施投资引导基金，建设沿边跨境金融服务中心。拓宽中小企业融资渠道，为23家工业企业发放“惠企贷”，放款3.34亿元，帮助企业争取上级扶持资金1.28亿元。推进农村金改，促进农村金融服务便利化，农村互联网金融融资超过1.6亿元。

（四）做优平台促开放。充分利用东兴试验区、构建开放型经济新体制试点试验城市、跨境合作区、沿边金融综合改革试验区等国家级平台政策资源，统筹推进跨境劳务合作和居民登记管理制度、跨境旅游合作等改革试点工作，在全区率先启动“大部门制、大警种制”改革。抓好边民互市贸易转型升级发展，被国务院督查组推荐为全国典型经验做法。在全国率先启用边民互市“一指通”系统，边境贸易成交额287.9亿元，增长23.4%。成功举办首届中越跨境经济合作论坛暨东兴试验区推介会、中越东兴-芒街商贸•旅游博览会、中国广西-越南广宁青少年友好交流活动等商贸文化交流活动。跨境合作区建设取得突破性进展，中越北仑河二桥竣工，国门楼封顶，友好大道、沿河大道、跨越大道等道路贯通，标准厂房、罗浮西路等一批重点项目加快建设。中越峒中-横模口岸桥开工建设。广西首家出口监管仓落户我市，东兴口岸通关人次首破700万。

（五）狠抓创新增动能。实施发明专利倍增计划，全市每万人口发明专利拥有量2.57件，增长84.9%。抓好创新创业工作，高新区形成云朗科技园、IT小镇门户、桃花湖新业态孵化园三大特色园区，吸引高科技研发企业、科技型生产企业入驻，明华、榕华被认定为国家级众创空间。防城港保税物流中心获自治区批复，东湾物流园成为全国首批国家级示范物流园区。防城港至北京、上海、沈阳等主要城市铁路冷链专列实现常态化运行，大西南出海大通道多式联运工程列入国家重点培育项目。推动电子商务发展，新增电商企业385家，实现销售额35.1亿元，增长75.5%。发展跨境游等特色旅游，全年接待国内外游客1580多万人次，增长16.4%，接待边境游人数17.5万人次，增长16.9%。

（六）提质增效强三农。实现农业总产值136.1亿元，增长4%。启动21个现代农业示范区创建工作，港口红树林海洋渔业示范区被认定为自治区级示范区。开展特色产业“10+3”行动，新种特色水果1万亩，面积达17.2万亩；发展牛大力、铁皮石斛等特色中药材2万亩，优质谷、莲雾等富硒特色农产品2000多亩；创建农业部水产健康养殖示范场2家、广西畜禽现代生态养殖场9家，珍珠湾国家级海洋牧场示范区成功获批。累计发展林下经济183万亩。新增农民专业合作社236家、家庭农场56家，农业产业化龙头企业达33家。扎实推进生态乡村建设，投入7718万资金，完成村屯绿化3466个。

（七）建设新区拓空间。贯彻落实中央、自治区城市工作会议精神，全面启动海湾新区建设，着力打造我市未来政治文化中心、科技金融中心、现代服务集群中心及港航服务配套中心。投资150亿元，推进100个海湾新区基础设施项目。“五横五纵”主干路网建设加快，东湾大道、西湾大道、文昌大道竣工通车，海湾绿道一期工程投入使用。园博园、伏波大道、文昌大桥等项目前期工作基本完成。

（八）夯实基础增后劲。抓好防城至江山一级路、马路至峒中公路、江山环岛东路、滨海公路龙门大桥西引道等项目建设，开工扩建钦防高速公路。加快建设防城港北站“两站两场两路”，增开动车组至5对，年客运量突破200万人次。企茅大道等十大基础设施项目前期工作取得新进展。渔万港区403-407号泊位竣工，40万吨级码头及航道、401-402、513-516号泊位、马鞍岭旅游码头等项目有序推进，渔万港区粮食输送改造工程投入运营。新建标准化海堤9.2公里、河堤及护岸5公里，除险加固中小型病险水库37座，大垌水库主体工程基本建成。防城港天然气专供管道建成通气。市垃圾焚烧发电厂投入使用。城镇污水处理率、生活垃圾无害化处理率均突破85%。

（九）精准施策抓脱贫。投入11.6亿元，实施脱贫攻坚“十五大”行动。强化挂钩帮扶、结对帮扶，全面完成精准识别建档立卡。出台19个脱贫配套政策，5125户18535人农村贫困人员纳入低保范围。产业扶持5996户贫困户，产业扶贫覆盖率达71.9%。加快异地扶贫搬迁，改造农村危旧房4093户，建成通村屯硬化道路417公里、便民候车亭20个。完成农村饮水安全工程237处，解决3688名贫困人口饮水问题。实现8706人脱贫、32个贫困村摘帽。

（十）千方百计惠民生。投入教育经费14.5亿元，加快教育攻坚。抓好132个项目建设，市理工学校、五中、六中、文昌小学、珍珠小学等一批学校基本竣工，北部湾高中开工建设，建成茅岭等4个乡镇中心幼儿园，基本实现乡镇学前教育全覆盖。推进一批农村义务教育学校增建扩容，港口区义务教育均衡发展通过国家验收。推进卫生攻坚，启动国家第四批城市公立医院综合改革试点，全市8家医院实现药品零差价。完善计划生育服务管理，落实全面二孩政策。创建国家公共文化服务体系示范区，成功举办国际海上龙舟节、东盟足球四国赛、中越沿边自行车赛、“春雨工程”走进防城港等一系列活动。启动国家社会保险标准化建设“先行城市”试点。新增城镇就业1.9万人，农村劳动力转移就业1.2万人。开工棚改项目7623套，基本建成2200套。民族团结进步模范市创建通过自治区验收。强化食品药品监管，食品安全形势稳定向好。落实安全生产监管责任，成功处置4·16东湾石油液化气泄漏事件。加强信访工作，成立市仲裁委员会，多元化纠纷解决机制不断完善。促进军民融合发展，摘取全国双拥模范城“四连冠”。推进平安防城港建设，强化边海防管控，严厉打击各类违法犯罪，人民群众安全感进一步增强。

此外，司法、侨务、人防、消防、保密、统计、税务、质监、供销、粮食、通信、海关、检验检疫、海事、气象、地震、水库移民、农机、宗教民族、社会科学、决策咨询、地方志、档案、法制、妇女儿童、老龄、残疾人、红十字、机关事务等工作取得新进步，工会、共青团、妇联等群团组织在经济社会发展中发挥了重要作用。全市边疆稳定，民族团结，社会和谐，人民安居乐业！

一年来，我们注重加强政府自身建设，扎实开展“两学一做”学习教育，狠抓全面从严治党和作风建设，认真履行“一岗双责”，严格落实中央“八项规定”和国务院“约法三章”，健全厉行节约机制，重点领域审计监督和行政监察取得新成效。深化“放管服”改革，全部取消非行政许可审批事项，在全区率先全面公布权力清单和责任清单。抓好全国商事制度改革试点，在全区率先实施企业名称自主选择登记和企业简易注销登记制度。市本级41%的审批事项在窗口办结，审批事项日结率达70.8%，每万人办件量稳居全区前列。严格依法行政，自觉接受人大和政协监督，人大代表建议和政协委员提案办复率100%。

各位代表！过去一年，我们取得的成绩来之不易。这是自治区党委、政府和市委正确领导的结果，是市人大、市政协监督支持的结果，是全市人民团结奋斗的结果。在此，我代表市人民政府，向全市各族人民，向我市驻军、武警官兵和中直、区直单位，向各民主党派、工商联、无党派人士、人民团体，向关心支持防城港发展建设的港澳台同胞、海外侨胞及海内外朋友们，表示崇高的敬意和衷心的感谢！

各位代表！一年的成绩铸就了新的起点，但前进的道路上还有不少困难和挑战。我市经济增长内生动力依然不足，企稳趋好的可持续性还不够强；工业投资和民间投资增长放缓，港口生产增速回落；现代服务业发展滞后；城市功能配套不够完善；脱贫攻坚任务依然繁重，教育卫生等短板依旧突出；政府效能建设有待进一步加强。我们必须以直面困难的勇气、敢于担当的精神，用新理念新方法认真加以克服和解决。

二、2017年工作安排

今年是实施“十三五”规划的重要一年，是供给侧结构性改革的深化之年，做好今年的政府工作意义重大。根据中央、自治区和市委的部署要求，今年政府工作的总体要求是：全面贯彻党的十八大和十八届三中、四中、五中、六中全会以及中央和全区经济工作会议精神，深入贯彻习近平总书记系列重要讲话精神，紧紧围绕市第六次党代会部署，统筹推进“五位一体”总体布局，协调推进“四个全面”战略布局，坚持稳中求进工作总基调，牢固树立和贯彻落实新发展理念，适应把握引领经济发展新常态，以推进供给侧结构性改革为主线，加快构建边海开放型经济新体制，全力推进东兴试验区、跨境经济合作区、沿边金融综合改革试验区建设，深入实施“四大攻坚”，促进经济平稳健康发展和社会和谐稳定，为着力营造“三大生态”、率先实现“两个建成”打下坚实基础，以优异成绩迎接党的十九大胜利召开。

根据总体要求，今年经济社会发展的主要预期目标是：地区生产总值增长9%；财政收入增长6%；固定资产投资增长11%；规模以上工业总产值增长14%；港口货物吞吐量增长7.5%；社会消费品零售总额增长10%；外贸进出口额增长14%；城镇居民人均可支配收入增长9%；农村居民人均可支配收入增长10%；居民消费价格指数控制在3%以内；城镇登记失业率控制在4%以内；万元地区生产总值能耗等指标控制在自治区要求范围内。

要完成上述目标任务，重点抓好以下工作：

（一）扭住项目建设不放松，积极扩大有效投资

围绕“边海经济带工程包”项目计划，发挥投资的关键作用，夯实经济社会发展基础。

强化重大项目建设。深入推进“边海经济带工程包”建设，争取新开工项目300个，再谋划一批新项目。开工建设企沙至茅岭公路、国门大道扩建、园博园、高新区基础设施项目。加快建设核电二期、钢铁基地、海工装备产业园、科元新材料、中海油LNG储运等一批重大项目，抓好沿海铝工业基地、新能源汽车产业园、支线机场、国际邮轮母港等项目前期工作，完善“四个一批”项目滚动机制，再掀项目建设新高潮。

强化项目建设保障。加强项目用地保障，做好土地收储，推进连片征地，妥善做好被征地群众社会保障和安置就业。抓好“旱改水”和耕地表土剥离利用，推进工业用地供地政策试点，推行新增耕地指标有偿使用制度。创新项目投融资机制，改革完善专项资金安排，激活民间投资，再推出一批PPP项目。

强化项目引进。创新招商方式，采用大数据应用等新型招商手段，以商招商，以企引企，不断提高招商引资的效率和质量。开展项目策划包装，落实项目共享机制，努力引进一批产业关联度高、科技含量高、带动能力强的好项目。完善招商工作机制，抓好签约项目的跟踪服务，强力推动项目早落地、早投产、早见效，形成“大项目顶天立地、中小项目铺天盖地、创新型项目开天辟地”的良好格局。

（二）大力发展实体经济，推动产业转型升级

深入实施创新驱动战略，加快发展新产业、新技术、新业态，促进经济结构转型和实体经济升级。

做强临港工业。抓住钢铁、有色金属、粮油等大宗商品市场回暖机遇，推动临港产业扩大规模。依托龙头企业带动，壮大“六大支柱”产业。服务好钢铁项目全面开工建设。抓好红沙核电二期、盛隆技改等项目建设。加快推进易高油砂、神雾新材料、中储能钒钛新材料、智能化麦芽、金川下游配套等项目建设。整合提升企沙工业区和大西南临港工业园，加快形成千亿元产业园区，打造成为广西北部湾地区重要新材料产业基地，努力创建国家级经济技术开发区。

提升现代服务业。创新服务业供给方式，破解制约服务业发展的瓶颈。抓好防城港国际航运服务中心等7个自治区级现代服务业集聚区建设。争取增开防城港至成都、石家庄、郑州、济南等城市铁路冷链专列，打造中国-东盟冷链物流集散基地。加快保税物流中心和综合保税区申报。发展多式联运，加快建设防城港集装箱办理站，推动东湾物流园“散改集”。参与中国-东盟信息港建设，创建国家级跨境电商试验区。加快边民互市贸易转型升级，延长加工贸易产业链。深入实施“引金入港”战略，引进更多银行、保险、证券等金融机构进驻。抓好跨境旅游合作区和边境旅游试验区申建工作，建设边关风情旅游带。推进国家全域旅游示范区和广西特色旅游名县建设，抓好白沙湾旅游接待基地等旅游项目，发展休闲游、养生游、工业游等特色品牌旅游。加强冬季旅游宣传推介，改变我市旅游“夏热冬冷”窘局。

注重发展非公经济。落实中央、自治区各类帮扶、奖励政策，支持民营企业参与政府投资项目和重大基础设施项目，参与发展市政公用事业，支持民办学校、民办医院健康发展。对符合发展要求的企业加大规划和土地支持，保障民营企业用地需求，搭建政企交流平台，营造公开透明、平等竞争的市场环境，聚力推动民营企业大发展。

发展现代特色农业。推进农业供给侧结构性改革，提升农业竞争力。把增加绿色优质农产品供给放在突出位置，狠抓农产品标准化生产、品牌创建。继续实施“10+3”特色产业行动计划，抓好现代特色农业示范区创建，发展“五金五红”等一批特色农产品，推进“双高”糖料蔗基地建设。加快渔港建设，发展远洋渔业，抓好离岸深水抗风浪网箱养殖及岸上工厂化养殖等现代设施渔业，加快推进珍珠湾国家级海洋牧场示范区建设。发展林下经济，优化林下种养结构。推进农业“六大工程”，高标准建设基本农田。实施农业龙头企业和农民专业合作社成长计划，加快土地有序流转，实现集约化规模化经营，抓好农产品加工和流通，培育发展休闲农业，推进农业“接二连三”融合发展。

发展壮大县域经济。实施大县城战略，坚持产业兴县、特色强县，引导县域错位发展、多元发展。上思县要打好生态旅游牌，深入推进脱贫攻坚战，提升制糖、水泥建材、木材加工三大传统产业，培育肉牛加工和制药两大新支柱产业，壮大生态休闲旅游优势产业，打造成为生态休闲旅游名县。东兴市要按照“前岸中区后城”发展模式，依托试验区等开放平台，发展贸易、加工、旅游、金融、电商、物流六大跨境产业，实现通道经济向产业经济转型升级，加快打造宜居国门城市。港口区要发挥边海经济带主战场、主力军作用，全力服务重大项目建设，壮大钢铁、能源、粮油加工、高新技术等产业，建设广西一流经济强区。防城区要加快促进临港工业配套产业的发展，统筹推进特色优势农业、现代服务业发展，建设现代服务业集聚区，加快建成国家生态文明示范区。

（三）推进基础设施攻坚，打造立体交通网络

围绕打造区域性国际枢纽门户城市目标，打好基础设施建设攻坚战，增强发展综合承载能力。

抓好港口建设。推进40万吨级码头及航道、东湾航道、企沙港区南作业区一期航道等重点港口项目前期工作，加快渔万港区501号和513-516号泊位、马鞍岭1、2号旅游码头、钢铁基地专用码头、渔万港区第五作业区航道、船舶溢油应急设备库等续建项目建设。推进渔万港区401号泊位、企沙港区潭油作业区3、4号泊位、榕木江西港点1-6号泊位、防城港4-7号锚地、渔万港区511、512号泊位等新开工项目建设。

加快重大交通设施建设。开工建设防东铁路，抓好防城港经上思至崇左、皇城坳至茅岭、云约江和潭油作业区等铁路项目规划建设。加快上思至东兴高等级公路、国道219峒中至东兴沿边公路、东湾跨海大桥、通用机场等项目前期工作，全力推进钦防高速改扩建工程、长途汽车客运站和公交站等项目建设，防城至江山一级路、马路至峒中公路、江山半岛环岛公路要建成通车，打造“港铁公机”立体交通枢纽。

（四）深化供给侧结构性改革和关键性改革，增强发展活力动力

按照统筹推进、重点突破的要求加快改革步伐，更好发挥改革牵引作用。

加快“三去一降一补”。坚持问题导向，落实各项措施，推进去产能、去库存、去杠杆、降成本、补短板五大任务取得实质性进展。去产能方面，深化国有企业改革，引导“僵尸企业”平稳退出，推动低端低效产能转型提升，发展一批中高端产能。去库存方面，推进“以购代建”和保障性安居工程货币化安置相结合模式，推进农民工市民化，促进存量商品房销售，实现销售150万平方米以上。实施房地产品质提升和供给结构调整计划，发展商业办公、旅游休闲、健康养老地产，打造中高端地产综合体，新开工50万平方米以上。去杠杆方面，强化政府债务规模控制和限额管理，抓好民间融资动态监测和风险预警，积极应对和妥善处置各类金融风险。降成本方面，深入落实自治区“41条”降成本政策措施，抓好政府性融资担保公司运营，继续开展“惠企贷”融资服务，推进电力直接交易，打好降低企业交易、人工、社保、财务、物流成本“组合拳”，力争今年企业综合成本下降5%以上。补短板方面，加快脱贫攻坚，提升教育卫生等公共服务水平，增强科技创新能力，加强基础设施薄弱环节，缩小城乡、区域发展差距，补齐全面小康“短板”。

推进一批重点领域改革。推进大部制改革和执法体制改革，争取设立口岸管理委员会，筹建市场监督管理局，深入推进公安局“大部制”改革。开展相对集中行政许可权改革试点，组建市行政审批局，完善“前置改后审、串联改并联、零散改集中”行政审批模式，强化政府部门清单制度管理。抓好跨境劳务合作、边民互市贸易转型升级改革试点。推进农地“三权分置”改革，加快国有林场、集体林权、农村金融、供销社等改革。加强改革协同，把责任压实、要求提实、考核抓实，让广大人民群众获得更多的改革红利。

（五）主动融入“一带一路”，加快发展开放型经济

充分发挥开放平台作用，主动服务“一带一路”“三大定位”等国家战略，推动对外开放再上新水平。

加快开放平台建设。全面推进构建开放型经济新体制四大改革和六大探索工作，形成在全国可复制可推广的“防城港经验和模式”。加快东兴试验区开发开放，抓好财税金融、跨境贸易、跨境劳务、跨境旅游、跨境电商等领域创新，扩大先行先试成果。大力推进中越跨境经济合作区纳入国家“一带一路”重大标志性工程清单，建成东兴口岸二桥综合服务区国门楼、标准厂房、罗浮西路等一批基础设施和产业项目，规划建设“一区两园”，实施精准招商，推动产业加快集聚。推进沿边金融综合改革试验区建设，拓展人民币与越南盾可兑换业务，探索建立人民币回流机制，布局建设股权产权交易中心、商品交易所，打造中国-东盟跨境金融中心。

推动口岸开放升级。深化口岸通关便利化改革，拓展防城港口岸、云约江南作业区、中电码头、企沙口岸开放范围。申报禽畜、肉类产品、生猪活体进口等指定口岸，建设进口肉类无害化处理设施。推动峒中口岸升格，加快建设里火互市区、北仑河二桥开放通道，完善陆运电子口岸。建设防城港旅检新口岸，加快口岸联检大楼进驻工作。推行东兴口岸“两国一检”监管模式，建设“三个一”信息平台，完善国际贸易“单一窗口”。

加强对外合作。主动融入“一带一路”建设，构建我市参与国际合作的新格局。拓展与越南等东盟国家经贸往来和港口合作，参与中国-东盟信息港建设，谋划建设中•印尼经贸合作区。开展与欧美等国家先进产能合作。强化与西南中南腹地的互动发展，推进“无水港”建设，发展“飞地经济”。加强与广东、浙江等东部沿海城市合作，积极承接产业转移。深度参与中国-东盟博览会，升级打造中越东兴-芒街商贸•旅游博览会，形成与东盟经贸人文交流的防城港渠道。

（六）强力推进城市建设，打造生态宜居海湾城市

落实中央、自治区城市工作会议精神，加快城市新区建设，提升城市功能品质，构建城市发展新格局。

加快海湾新区建设。实施“强中心”战略，科学制定海湾新区发展规划。加快推进江山大道、文昌大道等城市道路建设，开工建设园博园，确保完成2018年广西第十三届园林园艺博览会承办任务。规划建设文昌大桥、防城江三桥、新区配套路网等海湾新区十大工程，落实新区配水厂、10kv架空线路入地改造工程等基础项目，推行地下综合管廊建设，配套完善各类通讯管线。推进学校、医院、公园等公共服务设施建设，提升海湾新区公共服务能力。抓好伏波大道、百里黄金海岸、北部湾创业投资大厦等项目融资，采取土地招商、PPP项目运作、设立城市发展基金等多种方式筹措新区建设资金。推动产城深度融合，建设集生产、服务和消费于一体的经济综合体，努力打造富有特色、最具幸福感的美丽宜居新区。

提升城市品质。落实“九城同创”工作措施，打造城市核心品牌。加快推进西湾人文生态绿色廊道、边海大道绿化亮化提升工程等项目建设，完成北部湾大道、西湾大道等一批绿道建设，构建环西湾靓丽景观带。抓好西岸公园、金花茶公园、蜈蚣岭公园等一批市民休闲场所建设。推进海绵城市建设，提升绿地渗水蓄水功能。抓好城市污垃设施建设，促进城市环境和谐发展。推动城市管理综合执法体制改革，加强市容环境管理监察。建立城市监控指挥中心，打造智慧城市网格化综合服务管理平台。合理增设公交线路，淘汰更新公交设施，让市民出行更便捷更满意。

（七）强化创新驱动，不断培育发展新动能

全面实施创新驱动发展战略，以提高自主创新能力为重点，完善科技创新体系，促进科技创新能力整体提升。

建设创新平台。推进防城港高新区升级成为自治区级高新区。加快云朗科技园、沙潭江生态科技产业园建设，打造集多种功能于一体的产业新城。利用IT小镇门户等创新创业服务平台，引进一批高成长型科技企业和具有原创技术及商业模式的源头企业，扶持建设2个以上重点示范众创空间。构建“互联网＋”创业网络体系，打造中国-东盟离岸创业基地。支持有条件的企业开展新型学徒制试点，建立工程技术研究中心、重点实验室、院士专家工作站、博士后工作站、产业技术创新战略联盟等创新平台。

培育创新主体。实施高新技术企业倍增计划，建立科技企业、项目数据库，培育发展一批科技型中小企业，新增自治区级高新技术企业3家以上。突出企业主体地位，建设防城港产业研究院，设立中国-东盟技术转移中心-防城港分中心，推动科技创新与支柱产业有机衔接。推进“产学研”结合，加强与高校、科研院所的战略合作。实施大学生创业引领计划、农村青年创业富民行动，吸引各类域外人才创新创业。

健全创新机制。加强科技计划设计，完善科技项目管理办法。出台一批具有突破性、带有导向性的扶持创新政策措施，促进科技成果转化和技术转移。设立种子基金、重大科技专项创业投资基金，建设科技股权众筹平台，吸引更多社会资金投入科技产业。落实知识产权战略行动和发明专利双倍增计划，提高专利申请总量和质量，培育发展一批拥有自主知识产权和知名品牌的企业。倡导创新创业文化，激发全社会创新创造创业的精气神。

（八） 坚持以人民为中心，持续保障和改善民生

加快民生事业建设，破解民生难题、回应民生关切、满足民生需求，努力扩大共享发展成果。

抓好脱贫攻坚。深入实施脱贫攻坚“十五大行动”，抓好脱贫攻坚考核、退出、评估三项工作机制，对精准识别建档立卡管理系统动态管理，对脱贫工作进行过程跟踪、动态监测以及成效评估，实施脱贫“双认定”机制，抓好移民搬迁工程，完成移民搬迁5762人，确保28个贫困村、13611个贫困人口脱贫出列，完成上思县脱贫摘帽。

抓好教育攻坚。年内要新开工教育攻坚项目60个，北部湾高中争取年内建成使用，努力打造成全国知名普通高中。深入推进义务教育均衡发展，继续实施“全面改薄”工程，用好1.6亿元教育专项资金，补好农村义务教育短板，东兴要通过国家验收，上思、防城要通过自治区验收。抓好科教园区建设，力争再引进一家高等院校落户。抓好海湾新区等重点发展区域的教育基础建设，大力引入社会资本，优化配置教育资源，培养和引进一批优秀校长和骨干教师，努力办好人民满意的教育。

抓好卫生攻坚。深化医药卫生体制改革，加快城市公立医院改革，抓好市、县、乡、村四级卫生计生服务体系建设。推进市中医院和市妇幼保健院迁建，加快东兴医院综合楼、防城妇幼保健院和一批乡镇、社区卫生服务设施建设。坚持计划生育基本国策，完善计划生育服务管理。实施第二轮防治艾滋病攻坚工程，加强公共卫生服务体系规范化建设，全面提高市民健康水平。

促进和扩大就业。健全城乡就业服务机制，探索“互联网+创业孵化基地”模式，加强创业孵化基地、农民工创业园和众创空间等创业服务平台项目建设，落实农民工创业担保贷款政策，改善创业环境，以创业带动就业，实现城镇新增就业1.2万人，农村劳动力转移就业1.2万人。

加强社会保障。实施好城乡养老、城镇低保、边民0-3公里补助、残疾人救助等社保惠民项目。推进“全民参保登记计划”试点。抓好城镇保障性住房建设和农村旧危房改造，提高城乡居民住房保障水平。加快建立统一的城乡居民基本医疗保险制度，发展补充医疗保险和商业健康保险，让城乡居民享有更加公平的医保权益。

发展文体事业。全面完成国家公共文化服务示范区创建任务。完善城乡社区公共设施，加快村级公共服务中心、“村村通”等广播电视覆盖工程项目建设。开展全民读书活动，加强国学教育，建设“书香港城”。强化历史文化名镇保护，推进企沙渔港、那良、江平、平福等古镇古村规划建设。抓好上思举重、东兴足球等训练基地建设，推动各类体育场馆向公众开放，促进游泳、乒乓球等传统体育发展。办好海上国际龙舟节、京族“哈节”、中国-东盟水上嘉年华等文旅节庆活动，争取举办国际马拉松、自行车、汽车拉力赛和全国帆船邀请赛。

营造山清水秀自然生态。实施生态经济重点工程，建立生态经济重大项目库。加强生态治理和保护，严格执行“水十条”“大气十条”“土十条”，落实“河长制”，抓好“蓝色海湾”整治工程，强化近岸海域、港口粉尘、建筑工地、河流湖库综合治理，抓好红树林及沿海生态环境修复。加强城市水生态文明建设，推进黑臭水体和城区污水直排口整治。加快江山、光坡、马路和叫安等10个乡镇污水处理厂建设，实现镇级污水处理厂全覆盖。完成农村垃圾治理两年攻坚任务，90%的村庄生活垃圾得到有效处理。调整树种结构，控制速生桉种植面积。落实中央环境保护督察反馈问题整改，加强北仑河口等重点区域的生态保护，坚决守护好防城港的青山绿水、碧海蓝天！

推进“美丽家园”乡村建设。巩固提升清洁乡村、生态乡村建设成果，推进宜居乡村建设，抓好产业富民、服务惠民、基础便民三个专项活动。提高村落规划和民居设计水平，加快农村改厕、改厨、改圈、公共照明、村级垃圾处理终端等基础设施建设。推进“一村一镇”示范工程，抓好首批四个示范乡镇和27个示范村建设，抓好乡土特色示范村建设，重点打造上思平江村、东兴竹山村、港口沙螺寮村和防城冲敏村等8个“生态宜居示范村屯”。

营造团结和谐社会生态。深入实施幸福社区“三网四联五达标”工程，抓好农村社区建设试点，增强社区自治和服务功能。改进信访调解工作，依法调处化解各种矛盾纠纷。健全应急管理体制机制，提高综合防灾减灾救灾能力。加强重点行业领域、重点场所专项治理，坚决防范较大以上事故发生。完善市、县、镇三级食品药品安全监管网络，争取创建广西食品安全城市。提升气象科技和服务水平，率先在全区基本实现气象现代化。深化平安防城港建设，严厉打击走私贩毒和各类违法犯罪活动，不断提高人民群众安全感。开展民族团结进步“七进”活动，推进京族聚居区率先建成小康社会，抓好全国民族团结进步模范市创建。做好国防动员、“双拥”、优抚安置等工作，巩固发展军政军民团结，维护边疆和谐稳定。

三、加强政府自身建设

按照营造风清气正的政治生态要求，奋发有为、勤政务实，努力打造廉洁高效法治政府。

深入推进依法行政。完善政府立法体制机制，抓好市政府行使地方立法权工作。加快城市综合执法体制改革，推行跨部门、跨领域综合执法，推进行政执法权向乡镇延伸，建立行政执法信息平台。加强产权保护，依法保护公民产权权益和创新收益，营造公平公正的社会环境。自觉接受人大法律监督、工作监督和政协民主监督，认真办理人大代表议案、建议和政协委员提案。

着力优化政务服务。加大“放管服”力度，完善政府清单管理制度，强化事中事后监管，抓好“双随机、一公开”工作。推进网上审批，提升运行效率。整合乡镇“七站八所”政务服务资源，实现政务服务便捷化，打通群众办事“最后一公里”。克服形式主义、官僚主义，不断提高政府执行力。

健全行政决策机制。推行教育卫生、环境保护等重大民生决策事项民意调查制度。建立行政决策咨询论证专家库。健全重大行政决策社会稳定风险评估机制，加强重大决策第三方评估。发挥政府法律顾问作用，健全公共法律服务体系。强化行政监察、审计监督和社会舆论监督。

营造良好从政环境。巩固“两学一做”学习教育成果，认真履行“一岗双责”，严格执行“八项规定”。强化重点领域和关键岗位廉政风险防控，规范权力行使，严格落实政府采购、工程建设招投标程序，加强经济责任审计。推进预决算公开，让公共资金在阳光下运行。建立健全激励机制和容错纠错机制，激发干部干事创业积极性。倡导勇于担当负责精神，克服“庸懒散软”作风，真干、实干、加油干，树立风清气正的良好政府形象。

各位代表！“风帆劲鼓逐浪行，勇立潮头谱新篇”，戮力同心、砥砺前行的防城港正迸发出新的活力。让我们紧密团结在以习近平同志为核心的党中央周围，在市委的坚强领导下，坚定信心、保持定力，锐意进取、奋发有为，为着力营造“三大生态”，率先实现“两个建成”目标打下坚实基础，以优异成绩迎接党的十九大胜利召开！
